# Supplementary material for: SCF (Fbxl17) ubiquitylation of Sufu regulates Hedgehog signaling and medulloblastoma development
Source: EMBO J. 2016 May 27;35(13):1400–16. doi: 10.15252/embj.201593374 (PMC4884786; doi:10.15252/embj.201593374)

SCF (Fbxl17) ubiquitylation of Sufu regulates Hedgehog signaling and medulloblastoma development

APPENDIX - Table of Contents

Additional Materials and Methods

Figure Legends: Appendix Figures S1 – S5

Additional References

Appendix Figures S1-S5

Appendix

Additional Materials and Methods

DNA constructs generation

A cDNA coding full-length *Fbxl17* was purchased from origene (RC228545). For generation of Fbxl17 deletion constructs the appropriate nucleotide fragments carrying a Myc tag at the N-terminus were amplified by polymerase chain reaction (PCR) and cloned into the pcDNA 3.1 (+) plasmid. Additionally, full-length Fbxl17 was 3x FLAG tagged at the N-terminus and cloned into the pcDNA 3.1 (+) vector. This construct was used as template to generate the Fbxl17 ΔF construct as follows: 5'-phosphorylated primers containing an XhoI restriction site at each 5'end were designed to flank the F-box domain of Fbxl17. The PCR product was DpnI digested to eliminate the paternal template and circularized using the T4 DNA ligase (New England Biolabs). Mutant plasmids were transformed into competent E. coli (DH5α gold cells, Bioline). N-terminus Myc-tagged Fbxl17 was cloned into the pQCXIP retroviral expression vector that was used to generate DAOY and PC3 cells stably expressing Fbxl17. Fbxl17 with a Myc tag either at the N-terminus or at the C-terminus, as well as HA-tagged Fbxl17 was cloned into the pBABE Puro retroviral expression vector and used to stably transduced *Ptch*$^{-/-}$ MEFs.

Full-length of human *Sufu* coding sequence (isoform 1, NM_016169.3, generously provided by Dr. Christian Siebold, Nuffield Department of Medicine, University of Oxford, UK) was HA-tagged at the N-terminus and cloned into the pcDNA3.1 (+) vector. N-terminus and C-terminus truncated constructs of Sufu were generated using the above mentioned tag and plasmid. HA-tagged Sufu mutant K257R, as well as all the serine and threonine mutants used in this study, were generated from the full-length construct using the QuikChange site-directed

mutagenesis kit (Stratagene). Additionally, HA-tagged Sufu WT and Sufu mutant S352F were cloned into the pBABE-Puro vector.

Human *Gli1* coding sequence (isoform 1, NM_005269.2) carrying a FLAG tag at the C-terminus was PCR amplified and cloned into the pCMV5 plasmid.

All the constructs were verified by sequencing. The oligonucleotides used for the generation of all the constructs used in this study are available upon request.

Plasmid and siRNA transfections

For plasmid transfection, polyethylenimine (PEI Max, Polysciences Inc.) was used for transfection of HEK293T, X-tremeGENE HP (Roche) for NIH3T3 and HeLa cells and Lipofectamine LTX with *Plus* reagent (Invitrogen) was used for transfection of *Ptch1*$^{-/-}$ and *Sufu*$^{-/-}$ MEFs according to the manufacturer's instructions. For co-immunoprecipitation assay, 48 hours post-transfection, cells were treated with the Nedd8-activating enzyme inhibitor MLN4924 (1μM, Millenium Pharmaceuticals) and collected after 5 hours.

For siRNA transfection, 30nM of siRNA duplexes were transfected in HeLa, PC3, *Ptch1*$^{+/+}$, *Ptch1*$^{+/-}$ and *Ptch1*$^{-/-}$ MEFs using Lipofectamine RNAi MAX (Invitrogen) and in DAOY cells using HiPerFect (Qiagen). Next day, a second round of transfection was performed, and the cells were harvested 24 hours after the second transfection. This protocol was used for all cell lines, except DAOY for which one round of transfection was applied and the cells were harvested after 24 hours. The siRNA duplexes used to target human *Fbxl17* transcript (*Fbxl17* siRNA 1 and 2) were described earlier (Tan et al, 2013). To target the expression of human *Sufu* and mouse *Fbxl17* transcripts, we used Ambion® *Silencer*® Select Pre-designed siRNA

oligonucleotides (Sufu siRNA 1 [s28521] and Sufu siRNA 2 [s28522]; mFbxl17 siRNA 1 [s78415] and mFbxl17 siRNA 2 [s78417], respectively, Life Technologies).

For combined DNA and siRNA transfection, HEK293T cells were transfected using Lipofectamine 2000 (Invitrogen) according to the manufacturer's specifications. Plasmids were delivered to the cells along with the siRNA duplexes in the first round of transfection

Lysate production, co-immunoprecipitation and Western blotting

For the production of whole-cell lysates, cells were lysed in lysis buffer (LB) containing 50 mM tris-HCl at pH 7.4, 1mM EDTA, 150 mM NaCl, 5 mM $MgCl_2$, 0.1% NP-40, 1% glycerol and supplemented with protease and phosphatase inhibitors. After 10 minutes of incubation on ice, the lysates were clarified by centrifugation for 10 minutes in a refrigerated microcentrifuge at 14,000 rpm (revolutions per minute). All the immunoprecipitations involving Flag-tagged proteins were performed as described previously (D'Angiolella et al, 2012). Flag/Myc-tagged Fbxl17 co-immunopurified was analysed using liquid chromatography tandem mass spectrometry as described below. For co-immunoprecipitation of HA- or Myc-tagged, the appropriate antibodies were incubated with whole cell lysates for 2 hours at 4ºC followed by the addition of Protein G agarose beads (Sigma-Aldrich) and incubation for 2 additional hours. For co-immunoprecipitation of endogenous proteins, the corresponding primary antibodies and whole cell lysates were incubated overnight at 4 ºC with rotation. The beads were washed three times with LB and eluted in 2X SDS sample buffer. The samples were boiled for 5 minutes at 90ºC prior separation on 4–12% Novex bis-tris gels (Life Technologies). Primary antibodies were diluted at 1:1,000 in blocking solution (5% nonfat dry milk in PBS with 0.1% Tween 20) and

incubated for 1 hour at room temperature. HRP-conjugated secondary antibodies (1:1,000; Life Technologies) or HRP-conjugated Protein A/G (1:5,000; Pierce) were used for protein detection.

Cell fractionation

Ptch1$^{-/-}$ MEFs stably expressing Myc-tagged Fbxl17 were grown to confluence, serum staved in serum-reduced medium for 24 hours and submitted to cell fractionation using standard procedures described previously (Mendez & Stillman, 2000). Western blotting detection of Lamin A/C and GAPDH was performed to assess the purity of the nuclear and cytoplasmic extracts, respectively.

Protein turnover

HeLa cells were transfected with either non-targeting or two different Fbxl17 siRNA duplexes for two consecutive days, and 24 hours later, cells were washed and treated with 50 ug/ml cycloheximide (CHX) to inhibit further protein synthesis. At the end of each time point, cells were harvested, and endogenous Sufu was detected by immunoblot using 3 μg of whole-cell lysate. Sufu mutant S352F along with siRNA duplexes were transfected into NIH3T3 cells and CHX treatment was performed 24 hours after the second round of siRNA transfection when cells reached confluence.

RNA isolation, reverse transcription and real-time quantitative PCR (RT-qPCR)

Total RNA was purified using RNeasy Mini Kit (Qiagen) and complementary DNA was synthetized from 1 μg of total RNA using Oligo(dT) primers and Superscript III reverse transcriptase (Invitrogen) following the manufacturer's specifications. Quantitative RT-PCR was performed using gene-specific primers and

SYBR® Green PCR master mix (Life technologies) on a 7500HT Real-time PCR System (Applied Biosystems). Fbxl17, GAPDH, Sufu, Gli1 and Bcl2 primer sequences were described previously (Liu et al, 2006; Tan et al, 2013; Yue et al, 2009). All samples were assayed in triplicate and normalized to GAPDH.

Generation of stable cell lines

Myc-tagged Fbxl17, HA-tagged Fbxl17 and HA-tagged Sufu retroviral plasmids or an empty backbone retrovirus (EV) along with pCMV-Gag-Pol vector and pCMV-VSV-G envelope-encoding vector were used for retrovirus production in HEK293T cells. Twenty four hours post-transfection, the growth medium was changed. Forty-eight hours later, supernatants were harvested and filtered through a 0.45-µm filter. Stably transduced PC3, DAOY cells *Sufu*$^{-/-}$ and *Ptch*$^{-/-}$ MEFs were generated by retrovirus infection at 1 multiplicity of infection (MOI), in the presence of 8µg/ml polybrene, and followed by puromycin selection (1µg/ml) for 3 days. Expression of the protein of interest was confirmed by Western blotting.

Immunofluorescence

Ptch1$^{-/-}$ MEFs transduced with either Myc-tagged Fbxl17 expression construct or an empty vector were seeded on poly-D-lysine coated coverslips and cultured for 72 hours. Cells were fixed for 15 min at room temperature in PBS with 4% formaldehyde, permeabilised with 0.5% Triton X-100 in PBS and blocked using 5% BSA in PBS with 0.1% Triton X-100. Fbxl17 was detected using anti-Myc antibody at a dilution of 1:2,000, and endogenous Sufu was stained using goat anti-Sufu antibody at a dilution of 1:100. Primary antibody incubation was performed overnight at 4ºC. Secondary antibodies Alexa Flour® 488 donkey anti-mouse (1:1,000; Life

Technologies) was incubated for 30 min at room temperature. Nuclei were counterstained using DAPI and the coverslips were mounted with Prolong® Gold antifade reagent (Molecular Probes, Life Technologies). Cells were imaged on a Zeiss Axiovert microscope (63X oil immersion objective lens).

Cell proliferation assay

PC3 and DAOY cells were seeded in 6-well plates and transfected with either non-targeting or two different Fbxl17 siRNA duplexes for two consecutive days, and 24 hours later, cells were transferred into 15-cm plates and allowed to grow for 10 days. For standard rescue experiments, Myc-tagged Fbxl17 full-length construct was re-introduced in the cells 24 hours after the second round of siRNA. *Sufu*$^{-/-}$ MEFs transduced with an empty backbone retrovirus along with *Sufu*$^{-/-}$ cells stably expressing Sufu WT or Sufu mutant S352F were seeded in 10-cm plates and allowed to grow for 5 days. Cell proliferation rate was assessed by cell counting in three independent experiments. Cell counts corresponding to Fbxl17 siRNA-transfected cells were expressed relative to those of non-targeting siRNA, whereas the cell number corresponding to *Sufu*$^{-/-}$ expressing Sufu WT or Sufu S352F were represented relative to the control cell line.

GCPs culture and proliferation assay

Cerebellar GCPs were prepared from 5 old mice as previously described and plated at a density of 8 X 10^5 cells/cm^2 on eight-well chamber slides coated with 1 mg/ml poly-L-lysine. For RNA interference, GCPs were transfected with HiPerFect transfection reagent (Qiagen, Hilden, Germany), and for plasmids with Lipofectamine 2000 (Thermo Fisher Scientific, Waltham, MA, USA), according to the

manufacturer's instructions. Cell proliferation was evaluated by BrdU detection (Roche, Welwyn Garden City, UK). Briefly, 24 hours after transfection the BrdU pulse (24 hours) was performed. Cells were then fixed with 4% paraformaldehyde and permeabilized with 0.2% Triton X-100, and BrdU detection was performed according to the manufacturer's instructions. Nuclei were counterstained with Hoechst reagent. At least 500 nuclei were counted in triplicate, and the number of BrdU-positive or BrdU/GFP positive nuclei was recorded (Argenti et al, 2005).

Luciferase reporter gene assay

Sufu$^{-/-}$ MEFs and MEFs stably expressing WT or S352F mutant Sufu were seeded in 6-well plates and co-transfected with 1 µg of the luciferase reporter plasmid 8 X GliBS-luciferase (Sasaki et al, 1997) together with 250 ng of the *Renilla* luciferase pRL-TK reporter (Promega). Cells were serum starved 48 hours post-transfection and Hh stimulation using SAG was initiated 24 hours later. Cells lysis and dual-luciferase measurement were performed 48 hours post-induction using the Dual-Glo luciferase assay system (Promega). All measured luciferase activities were normalized to pRL-TK.

shRNA lentivirus production and Fbxl17 knockdown

Endogenous Fbxl17 was knocked down using shRNA delivered to cells via a lentiviral system. The shRNA-expressing lentiviruses were generated as previously described (D'Angiolella et al, 2012). Expression arrest pGIPZ lentiviral vectors encoding a non-targeting shRNA (5'-TCTCGCTTGGGCGAGAGTAAG-3') or Fbxl17 shRNAs (5'-AGACAAGACCTATCAGTAA-3') were provided by the Target Discovery Institute High Throughput Core (University of Oxford, UK). Transduction

of DAOY cells with either non-targeting or Fbxl17 shRNA-expressing lentiviruses was carried out in the presence of 8µg/ml polybrene, followed by selection in growth medium containing 1µg/ml puromycin during 3-4 days.

Immunohistochemistry

All animals were transcardially perfusion-fixed under terminal anaesthesia with 0.9% heparinised saline followed by periodate lysine paraformaldehyde (PLP) containing only 0.025% glutaraldehyde (PLP_{light}) at the end of the experiment (10 week after tumour induction). The brains were post-fixed, cryoprotected, embedded in tissue-tek (Sakura Finetek Europe) and frozen in isopentane at -40˚C. Frozen, 10-µm thick, serial sections spanning the tumour in the cerebellum were cut from fixed tissue using a cryostat (Leica CM1900) and mounted onto gelatine-coated slides.

Immunohistochemistry was performed with antibodies against markers of DAOY cells (Vimentin) and proliferation (Ki67). Sections were counter-stained with 0.5% cresyl violet (Sigma Aldrich).

In each group of animals, tumour area per mm^2 of brain were quantified on four 10-µm-thick sections immediately adjacent to the injection site by drawing around the area of vimentin staining using Aperio ImageScope Software (Leica microsystem). Proliferative index was calculated by determining the percentage of Ki67 positive cells (brown) within the tumour from four brain sections spanning the tumor site.

Appendix Figure legends

Appendix Figure S1

A. **Detected posttranslational modifications of wildtype Sufu after immuno-purification and LC-MS/MS analysis.** Blue bars represent detected Sufu derived peptides. S352 is covered with multiple detections and modifications. Phosphorylated S352 was not detected. S346, S349 and T353 were found phosphorylated and the sites confirmed by presence of diagnostic fragment ions in the MS/MS spectra.

B. **Sufu degradation by Fbxl17 allows Gli dissociation for Hedgehog signaling activation.** Detection of Fbxl17 mRNA levels in *Ptch1*$^{-/-}$ MEFs transfected with non-targeting siRNA (Control) or two siRNAs against Fbxl17.

Appendix Figure S2. Fbxl17 mediated degradation of Sufu regulates Hh signaling and proliferation in prostate cancer cell (PC3).

A. Gli1 mRNA levels after transfection of PC3 cells with a non-targeting siRNA (Control) or two siRNAs to Fbxl17 (1) and (2). (mean ± SEM from 3 independent experiments, **p<0.005).

B. Gli1 mRNA levels in PC3 cells after transfection with an Empty Vector (EV) or a vector expressing Myc-Fbxl17. (mean ± SEM from 3 independent experiments, **p<0.005).

C. Cell proliferation of PC3 cells upon Fbxl17 depletion using two different siRNAs or upon reintroduction of a Myc-tagged Fbxl17 construct in Fbxl17-depleted PC3 cells (mean ± SEM from 3 independent experiments, *p<0.05; ***p<0.0005).

D. Sufu protein levels in PC3 cells treated as in C. Representative image of three independent experiments is shown.

E. Relative abundance of Sufu protein in PC3 cells treated as in C (mean ± SEM from 3 independent experiments, *p<0.05; **p<0.005).

F. Quantification of Fbxl17 mRNA levels in PC3 cells upon transfection with a non-targeting siRNA (Control) or two siRNAs to Fbxl17 (1) and (2).

Appendix Figure S3. Fbxl17 mediated degradation of Sufu regulates proliferation of prostate cancer cells (PC3)

A. Cell proliferation of PC3 cells transfected with non-targeting siRNA (Control), siRNA against Fbxl17 or a combination of siRNA targeting Fbxl17 and Sufu. (mean ± SEM from 3 independent experiments, ***p<0.0005; NS, non-significant).

B. Detection of Sufu protein levels treated as in A. Representative image of three independent experiments is shown.

C. Relative abundance of Sufu protein in PC3 cells treated as in A (mean ± SEM from 3 independent experiments, *p<0.05).

D. Quantification of Fbxl17 mRNA levels in PC3 cells upon Fbxl17 depletion using either a non-targeting siRNA (control) or two siRNAs to Fbxl17 (1) and (2) (mean ± SEM from 3 independent experiments, ***p<0.0005).

Appendix Figure S4. Fbxl17 mediated degradation of Sufu regulates proliferation of medulloblastoma cells (DAOY)

A. Relative abundance of Sufu protein in DAOY cells treated as in Fig 5A (mean ± SEM from 3 independent experiments, *p<0.05; **p<0.005; ***p<0.05).

B. Relative abundance of Sufu protein in DAOY cells treated as in Fig 5D (mean ± SEM from 3 independent experiments, **p<0.005; ***p<0.0005).

Appendix Figure S5. T1-weighted MRI assessing medulloblastoma tumour growth upon Fbxl17 silencing

A. Relative abundance of Sufu protein levels in DAOY cells transfected with Control shRNA or shRNA against Fbxl17 (mean ± SEM from 3 independent experiments, ***p<0.0005).

B. T_1-weighted magnetic resonance representative images taken at 10 weeks showing vessel permeability in rats injected with DAOY cells transfected with control shRNA or shRNA against Fbxl17. Scale bar: 5mm.

C. Bar chart showing gadolinium-enhanced T_1 hyperintensity (a surrogate marker of tumour vessel permeability) at 10 weeks in both groups. Animals were injected with DAOY cells stably expressing either control shRNA or shRNA against Fbxl17 (10,000 cells/1 µL). (mean ± SEM; n=5; two-way ANOVA, followed by unpaired t-test, *p<0.05, **p<0.01).

References

Argenti B, Gallo R, Di Marcotullio L, Ferretti E, Napolitano M, Canterini S, De Smaele E, Greco A, Fiorenza MT, Maroder M, Screpanti I, Alesse E, Gulino A (2005) Hedgehog antagonist REN(KCTD11) regulates proliferation and apoptosis of developing granule cell progenitors. *The Journal of neuroscience : the official journal of the Society for Neuroscience* **25:** 8338-8346

D'Angiolella V, Donato V, Forrester FM, Jeong YT, Pellacani C, Kudo Y, Saraf A, Florens L, Washburn MP, Pagano M (2012) Cyclin F-mediated degradation of ribonucleotide reductase M2 controls genome integrity and DNA repair. *Cell* **149:** 1023-1034

Liu G, Yuan X, Zeng Z, Tunici P, Ng H, Abdulkadir IR, Lu L, Irvin D, Black KL, Yu JS (2006) Analysis of gene expression and chemoresistance of CD133+ cancer stem cells in glioblastoma. *Molecular cancer* **5:** 67

Mendez J, Stillman B (2000) Chromatin association of human origin recognition complex, cdc6, and minichromosome maintenance proteins during the cell cycle: assembly of prereplication complexes in late mitosis. *Molecular and cellular biology* **20:** 8602-8612

Sasaki H, Hui C, Nakafuku M, Kondoh H (1997) A binding site for Gli proteins is essential for HNF-3beta floor plate enhancer activity in transgenics and can respond to Shh in vitro. *Development* **124:** 1313-1322

Tan MK, Lim HJ, Bennett EJ, Shi Y, Harper JW (2013) Parallel SCF adaptor capture proteomics reveals a role for SCFFBXL17 in NRF2 activation via BACH1 repressor turnover. *Mol Cell* **52:** 9-24

Yue S, Chen Y, Cheng SY (2009) Hedgehog signaling promotes the degradation of tumor suppressor Sufu through the ubiquitin-proteasome pathway. *Oncogene* **28:** 492-499

Appendix Figure S1

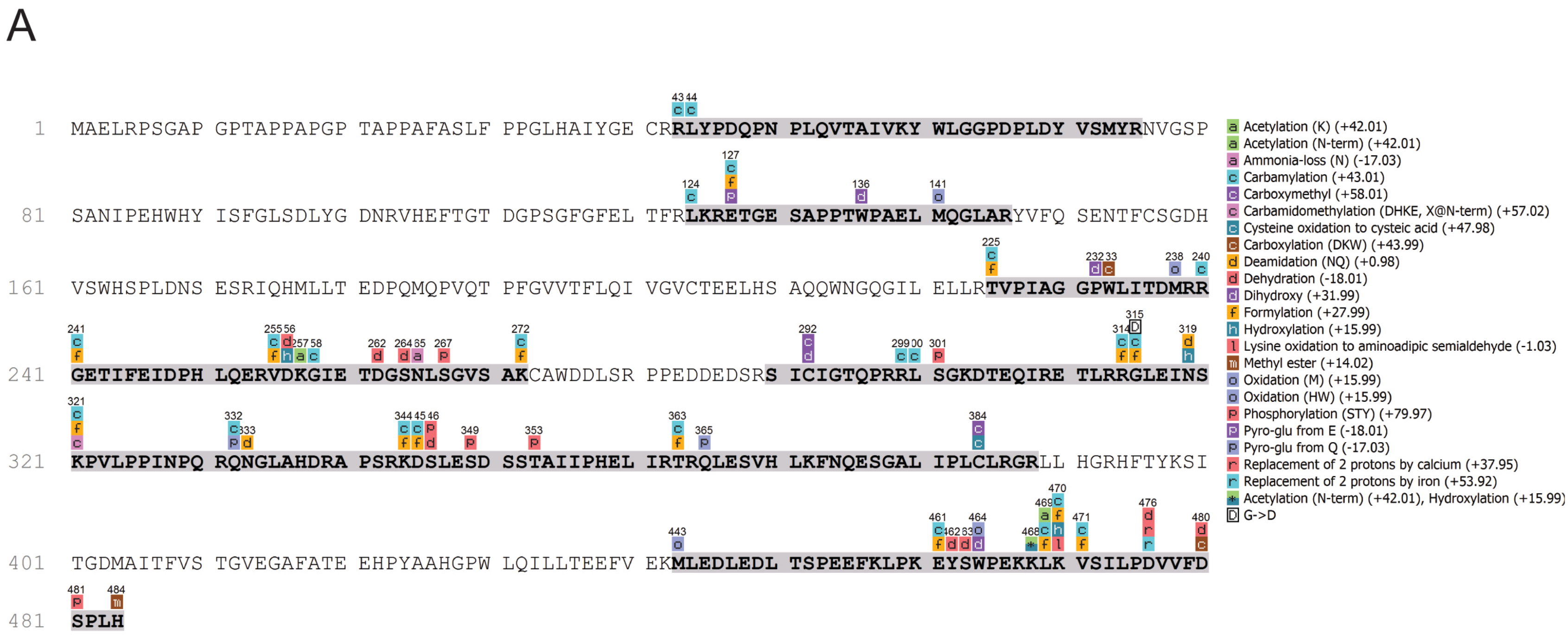

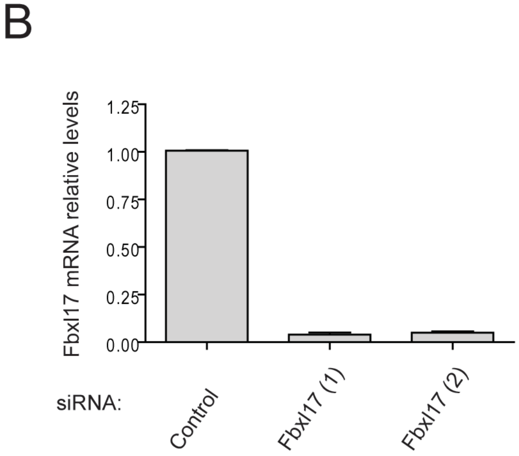

Appendix Figure S2

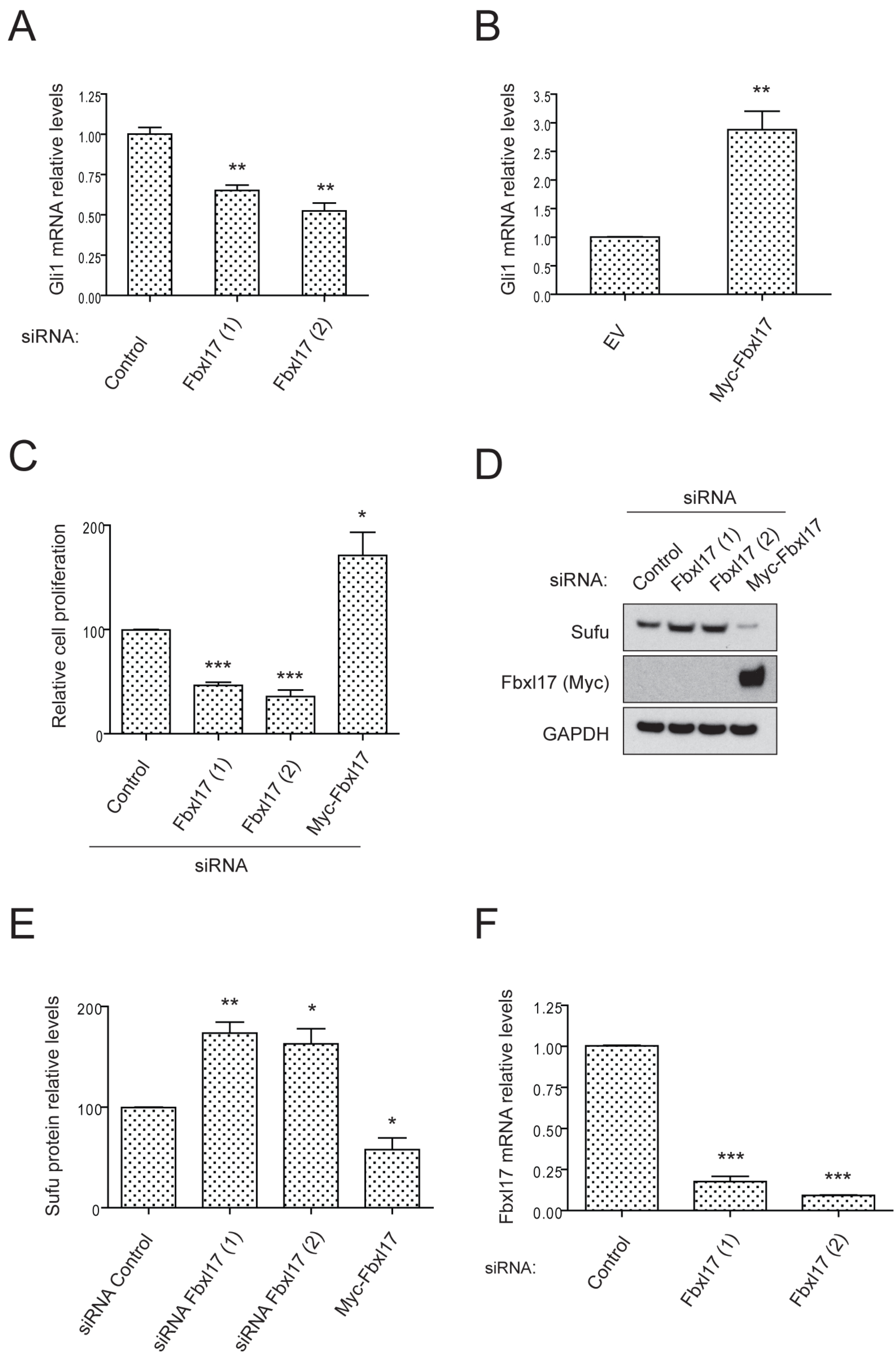

Appendix Figure S3

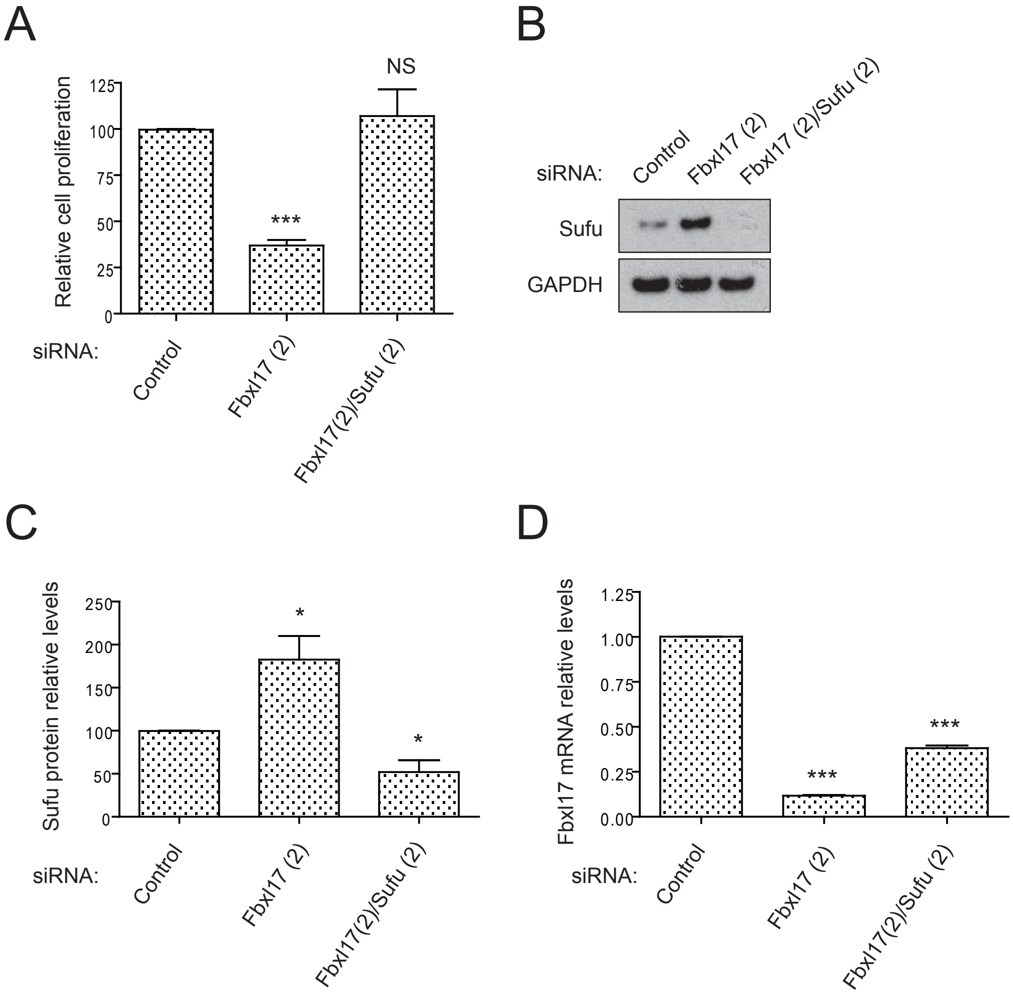

Appendix Figure S4

A

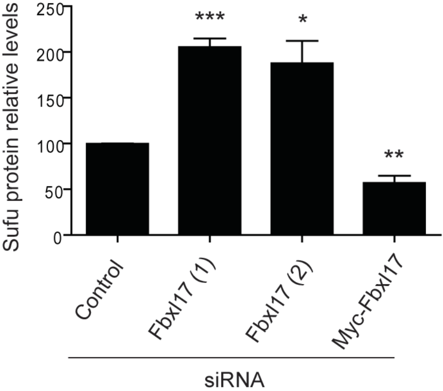

B

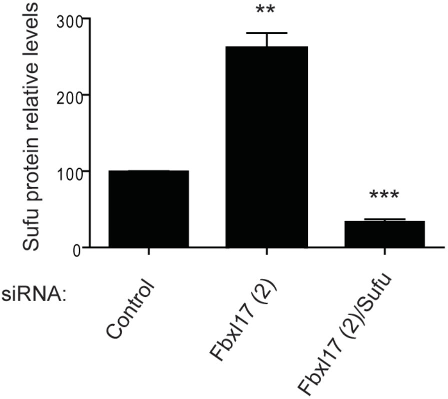

Appendix Figure S5

A

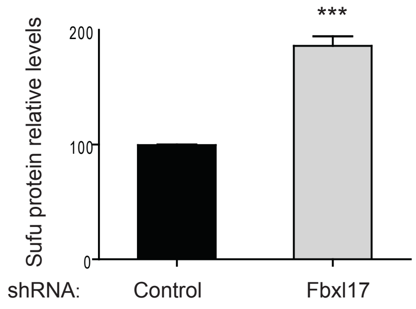

B

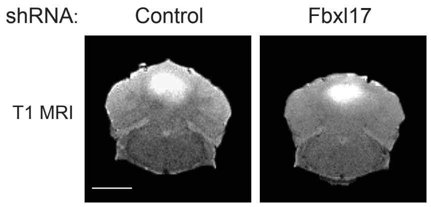

C

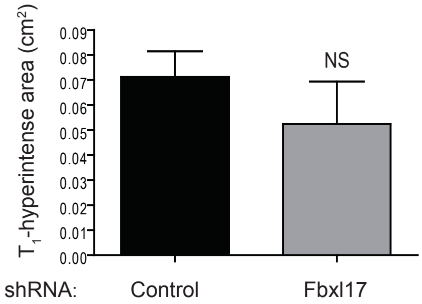

Supplement: Supplementary file 1 — Appendix [file EMBJ-35-1400-s001.pdf]
