# Supplementary material for: SCF (Fbxl17) ubiquitylation of Sufu regulates Hedgehog signaling and medulloblastoma development
Source: EMBO J. 2016 May 27;35(13):1400–16. doi: 10.15252/embj.201593374 (PMC4884786; doi:10.15252/embj.201593374)
Supplement: Supplementary file 2 — Expanded View Figures PDF [file EMBJ-35-1400-s002.pdf]

Expanded View Figures

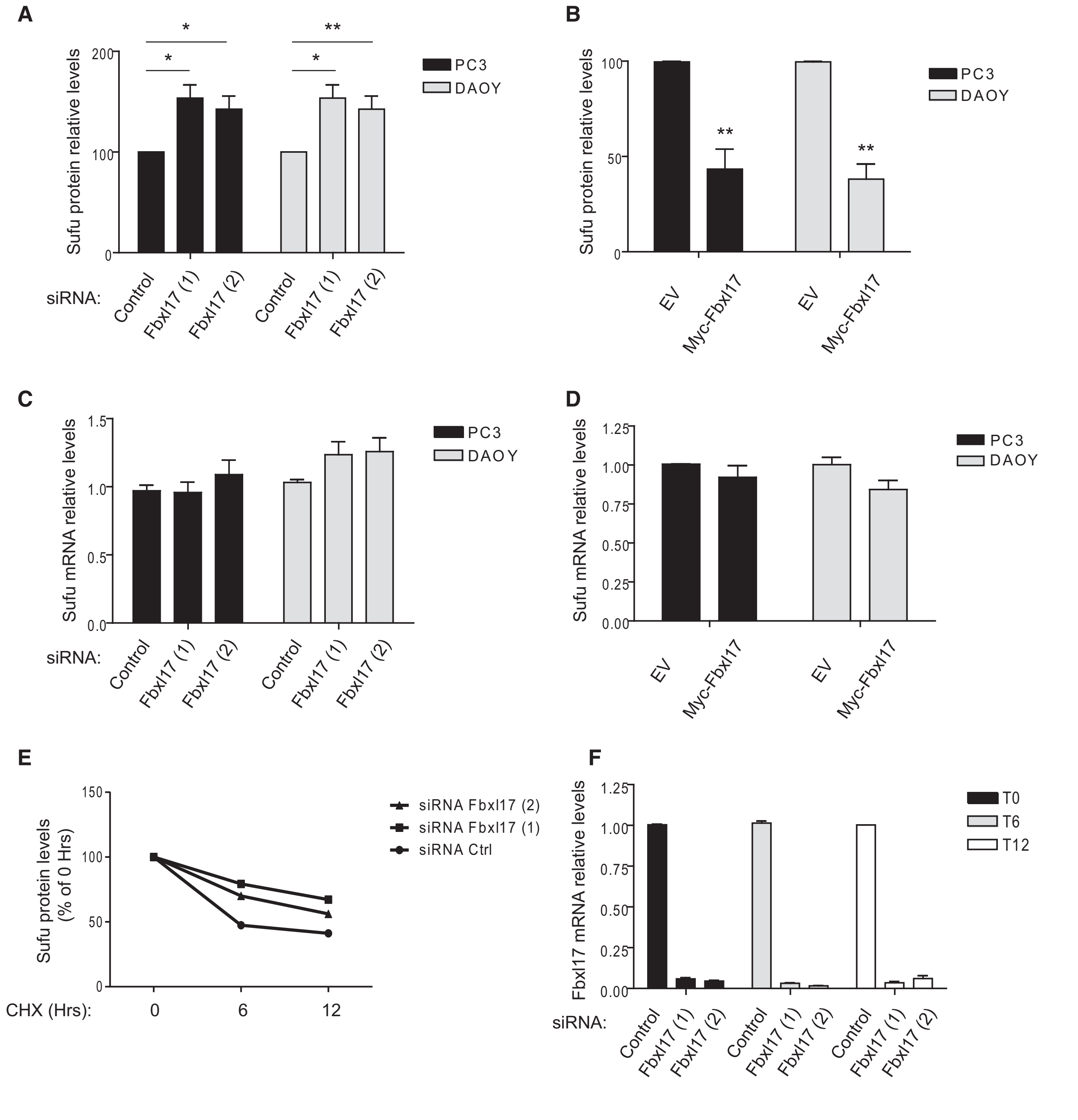

Figure EV1. Fbxl17 modulates Sufu protein levels, without affecting Sufu mRNA.

A Relative abundance of Sufu protein levels in PC3 and DAOY cells reported in Fig 1E (mean ± SEM from three independent experiments, *$P < 0.05$; **$P < 0.005$).
B Relative abundance of Sufu protein levels in PC3 and DAOY cells reported in Fig 1F (mean ± SEM from three independent experiments, **$P < 0.005$).
C Sufu mRNA levels in PC3 and DAOY cells transfected with either a nontargeting siRNA (Control) or two siRNAs to Fbxl17 (1) and (2) (mean ± SEM).
D Sufu mRNA levels after the transfection of the indicated cell lines with an empty vector (EV) or a vector expressing Myc-Fbxl17 (mean ± SEM).
E Relative abundance of Sufu protein levels in HeLa cells reported Fig 1G. Data are normalized relative to 0 h (Hrs) of CHX (cycloheximide) treatment.
F Fbxl17 mRNA levels in HeLa cells treated as in Fig 1G (mean ± SEM).

 © 2016 The Authors

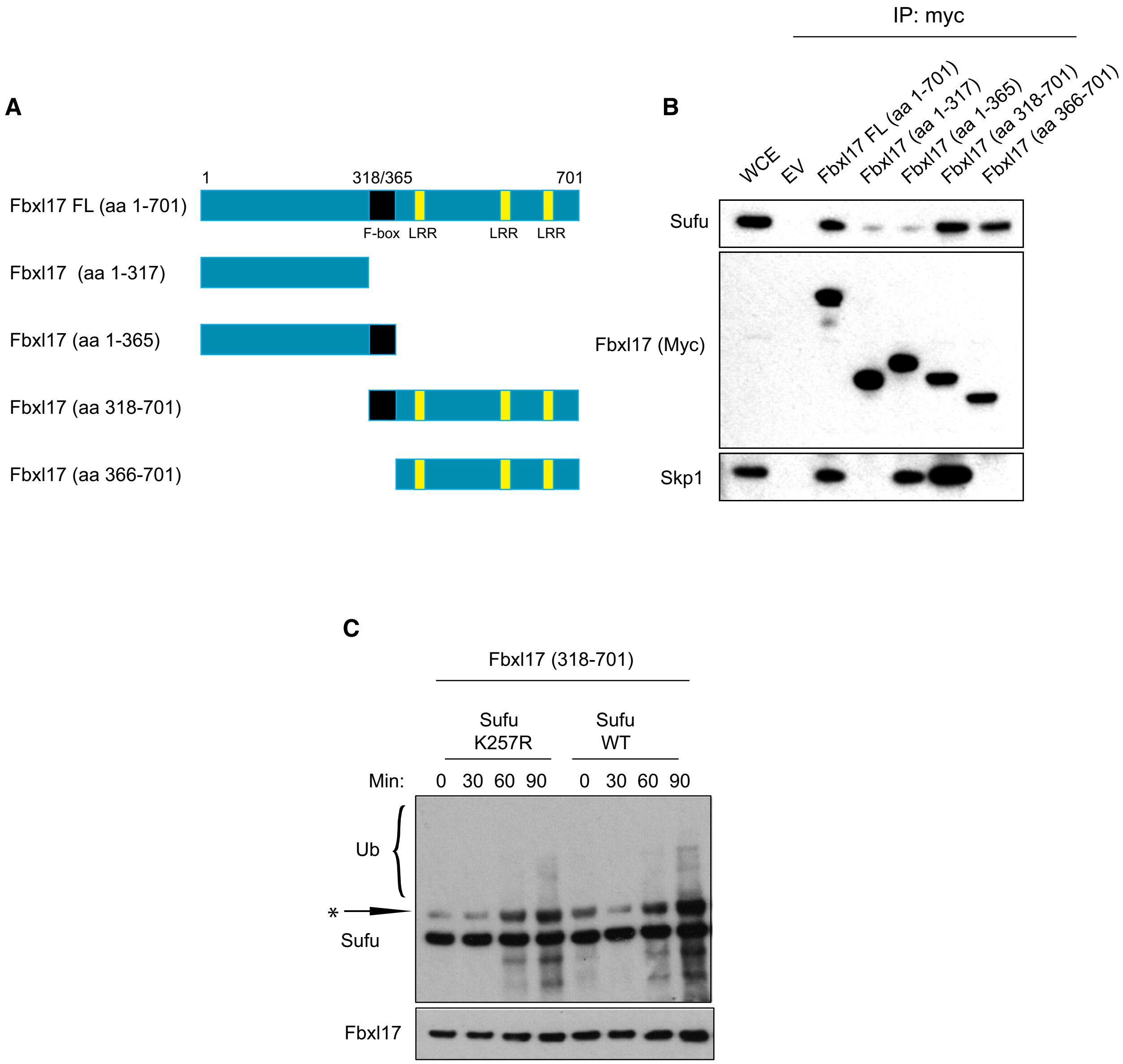

Figure EV2. Sufu polyubiquitylation requires interaction with the carboxyl terminus of Fbxl17.

A Scheme representing the mapping strategy to identify Fbxl17 region required for Sufu recognition. The F-box is highlighted in black and the leucine-rich repeats (LRR) in yellow.

B Detection of Sufu and Skp1 after immunoprecipitation of Myc-tagged Fbxl17-truncated fragments, as indicated. An empty vector (EV) was used as a negative control. HEK293T cells were treated with MLN4924 (2 μM) for 5 h prior to collection.

C *In vitro* ubiquitylation assay of Sufu WT and Sufu mutant K257R by Fbxl17-truncated version 318–701. All proteins were synthesized *in vitro* using a T7-coupled reticulocyte lysate system and incubated for the indicated times. Arrow indicates a nonspecific band detected in reticulocyte lysates.

© 2016 The Authors

Figure EV3. Systematic analysis of Sufu mode of interaction to Fbxl17.

- A Schematic representation of Sufu-truncated mutants used to identify Sufu region required for Fbxl17 recognition. The black square delimitates the minimal binding region 350–425.
- B Detection of Myc-tagged Fbxl17 after immunoprecipitation of HA-Sufu-truncated fragments, as indicated in (A). An empty vector (EV) was used as a negative control. HEK293T cells were treated with MLN4924 (2 μM) for 5 h prior to collection.
- C Detection of Myc-tagged Fbxl17 binding to HA-tagged Sufu deletion construct 350–425 in HEK293T cells. Transfection of an empty vector (EV) was used as a negative control for immunoprecipitation. Cells were treated with MLN4924 (2 μM) for 5 h prior to collection.
- D Detection of Myc-tagged Fbxl17 binding to immunoprecipitated HA-tagged Sufu wild type (WT), Sufu S352F, or Sufu S352D. An empty vector (EV) was used as a negative control. HEK293T cells were treated with MLN4924 (2 μM) for 5 h prior to collection.
- E Detection of Flag-tagged Gli1 and Myc-tagged Fbxl17 after immunoprecipitation of HA-tagged Sufu WT or Sufu S342/6A and S342/6D, as indicated. HEK293T cells were treated with MLN4924 (2 μM) for 5 h prior to collection.
- F Detection of Myc-tagged Fbxl17 after immunoprecipitation of the indicated HA-Sufu point mutants in HEK293T cells. Cells were treated with MLN4924 (2 μM) for 5 h prior to collection.

 © 2016 The Authors

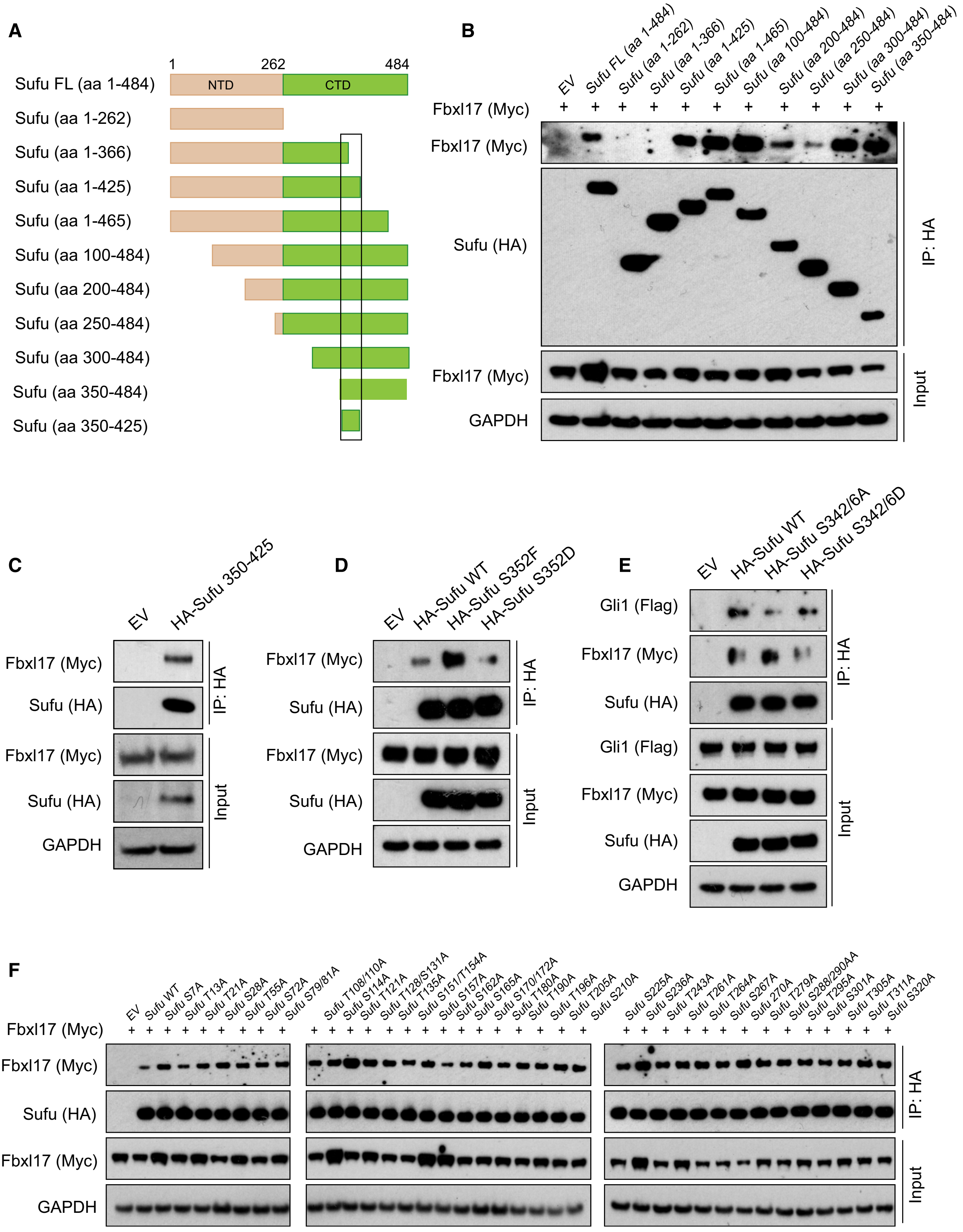
A
1
262
484
Sufu FL (aa 1-484)
NTD
CTD
Sufu (aa 1-262)
Sufu (aa 1-366)
Sufu (aa 1-425)
Sufu (aa 1-465)
Sufu (aa 100-484)
Sufu (aa 200-484)
Sufu (aa 250-484)
Sufu (aa 300-484)
Sufu (aa 350-484)
Sufu (aa 350-425)
B
EV
Fbxl17 (Myc)
Sufu (HA)
GAPDH
IP: HA
Input
C
HA-Sufu 350-425
D
HA-Sufu WT
HA-Sufu S352F
HA-Sufu S352D
E
HA-Sufu S342/6A
HA-Sufu S342/6D
Gli1 (Flag)
F
Sufu WT
Sufu S7A
Sufu T13A
Sufu T21A
Sufu S28A
Sufu T55A
Sufu S72A
Sufu S79/81A
Sufu T108/110A
Sufu S114A
Sufu T121A
Sufu T128/S131A
Sufu T135A
Sufu S151/T154A
Sufu S157A
Sufu S162A
Sufu S165A
Sufu S170/172A
Sufu T180A
Sufu T190A
Sufu T196A
Sufu T205A
Sufu S210A
Sufu S225A
Sufu S236A
Sufu T243A
Sufu T261A
Sufu T264A
Sufu S267A
Sufu 270A
Sufu T279A
Sufu S288/290AA
Sufu T295A
Sufu S301A
Sufu T305A
Sufu T311A
Sufu S320A

Figure EV3.

Figure EV4. Fbxl17 regulates Hh signaling in DAOY medulloblastoma cells.

A Gli1 mRNA levels in DAOY cells upon the treatment with SAG or cyclopamine. DMSO was used as a control vehicle (mean ± SEM from three independent experiments, $^{**}P < 0.005$; $^{***}P < 0.0005$).

B Gli1 mRNA levels in DAOY cells transfected with nontargeting siRNA (Control) or siRNAs against Fbxl17 (2) and treated with DMSO (mean ± SEM).

C Gli1 mRNA levels in DAOY cells transfected as in (B) and treated with SAG (mean ± SEM from three independent experiments, $^{*}P < 0.05$).

D Gli1 mRNA levels in DAOY cells transfected as in (B) and treated with cyclopamine (mean ± SEM).

E Gli1 mRNA levels in DAOY cells treated with control medium (−) or medium containing SHH ligand (+) (mean ± SEM from three independent experiments, $^{**}P < 0.005$).

F Detection of Sufu protein levels in DAOY in the presence of a nontargeting siRNA (Control) or two siRNAs to Fbxl17 (1) and (2), after the treatment with control medium (−) or medium containing SHH (Sonic Hedgehog) ligand (+).

G Relative abundance of Sufu protein levels detected in (F) (mean ± SEM from three independent experiments, $^{**}P < 0.005$; $^{***}P < 0.05$).

H Fbxl17 mRNA levels in DAOY cells upon the treatment with DMSO, SAG, or cyclopamine (mean ± SEM).

I Fbxl17 mRNA levels in DAOY cells after the treatment with control medium (−) or medium containing SHH ligand (+) (mean ± SEM).

© 2016 The Authors

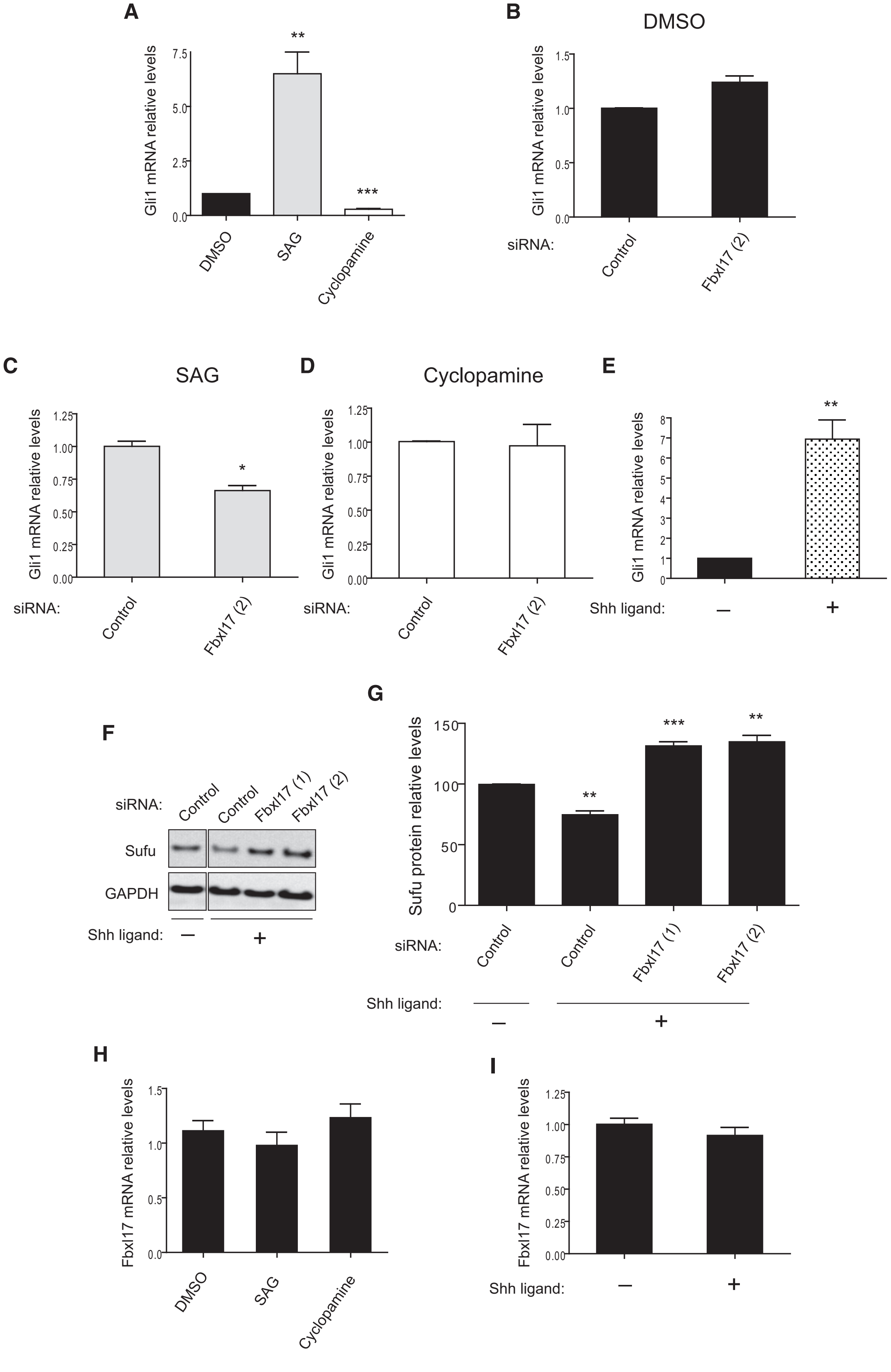
A
B
DMSO
C
SAG
D
Cyclopamine
E
F
G
H
I
Gli1 mRNA relative levels
siRNA:
Control
Fbxl17 (2)
Fbxl17 (1)
SAG
DMSO
Cyclopamine
Shh ligand:
Sufu
GAPDH
Sufu protein relative levels
Fbxl17 mRNA relative levels

Figure EV4.

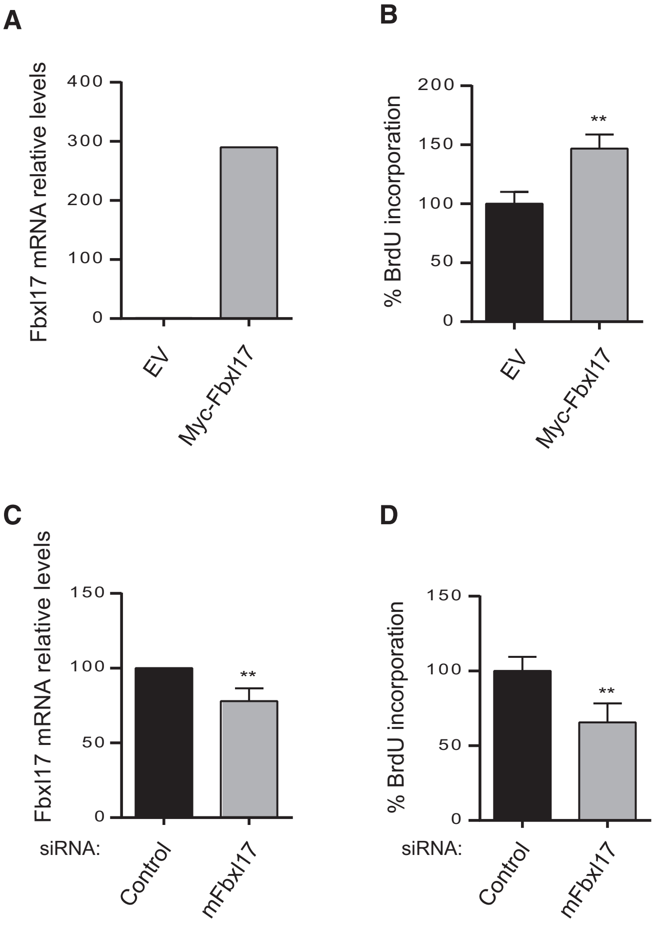

Figure EV5. Fbxl17 regulates the proliferation of granule cell progenitors (GCPs).

A Fbxl17 mRNA levels in GCPs after transfection with an empty vector (pCDNA) or a vector expressing Myc-Fbxl17 (Fbxl17) (mean ± SEM from three independent experiments, $**P < 0.005$).

B Cell proliferation of GCPs upon Fbxl17 overexpression (mean ± SEM from three independent experiments, $**P < 0.005$).

C Fbxl17 mRNA levels in GCPs transfected with either a nontargeting siRNA (Control) or siRNAs to Fbxl17 (mean ± SEM from three independent experiments, $**P < 0.005$).

D Cell proliferation of GCPs upon Fbxl17 depletion (mean ± SEM from four independent experiments, $**P < 0.005$).

Source data are available online for this figure.

© 2016 The Authors
