# Supplementary figures and images for: SCF (Fbxl17) ubiquitylation of Sufu regulates Hedgehog signaling and medulloblastoma development
Source: EMBO J. 2016 May 27;35(13):1400–16. doi: 10.15252/embj.201593374 (PMC4884786; doi:10.15252/embj.201593374)

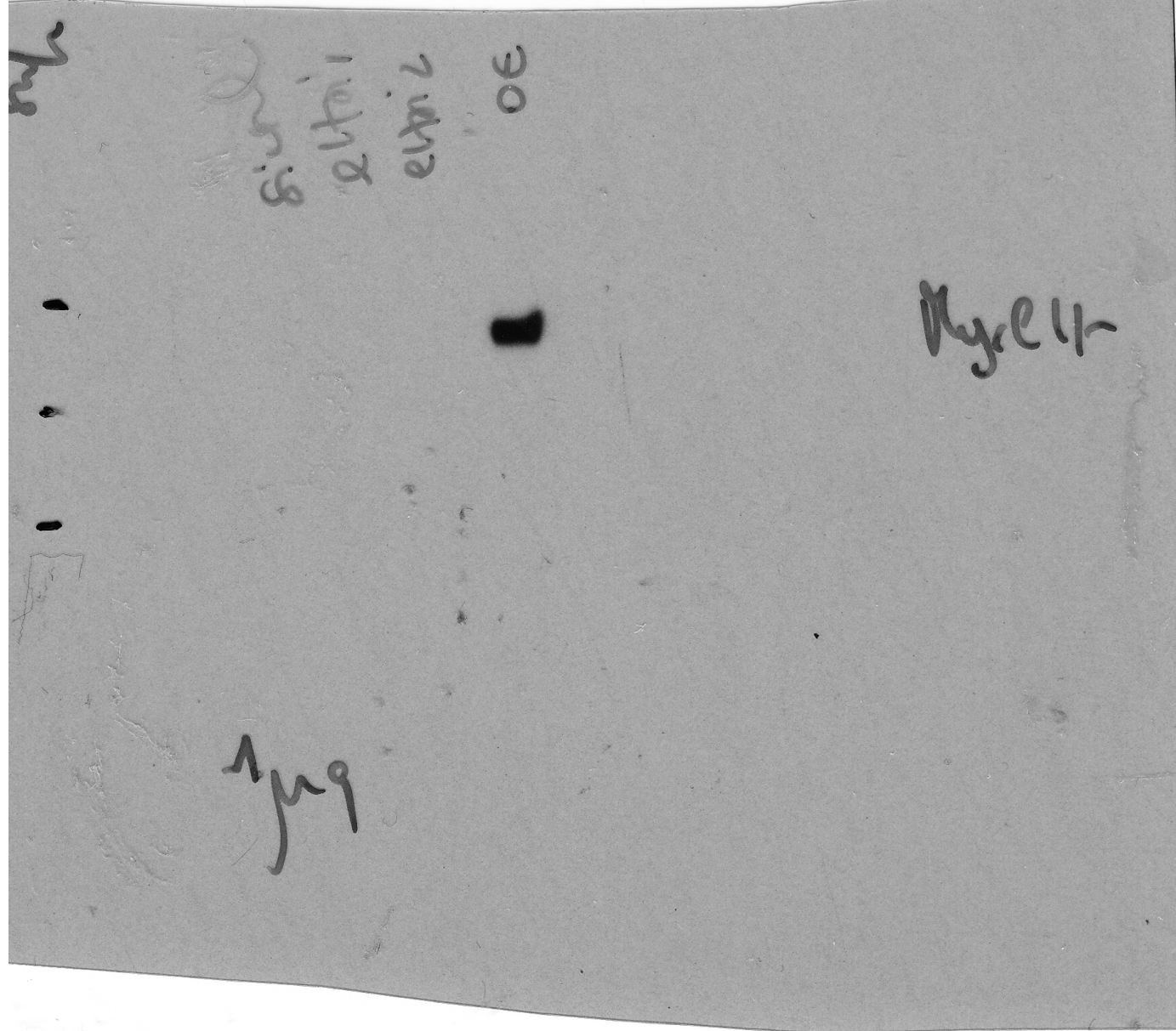

sh

8

eltai

eltai

oe

H

129

Sub

val  
litri  
litri  
oc  
val  
litri  
litri  
oc

GAPDH

PC 3

Supplement: Supplementary file 3 — Source Data for Expanded View [file EMBJ-35-1400-s003.zip › Fig_EV5_Source_Data.pdf]
