# Supplementary material for: SCF (Fbxl17) ubiquitylation of Sufu regulates Hedgehog signaling and medulloblastoma development
Source: EMBO J. 2016 May 27;35(13):1400–16. doi: 10.15252/embj.201593374 (PMC4884786; doi:10.15252/embj.201593374)
Supplement: Supplementary file 5 — Source Data for Figure 5C [file EMBJ-35-1400-s004.pdf]

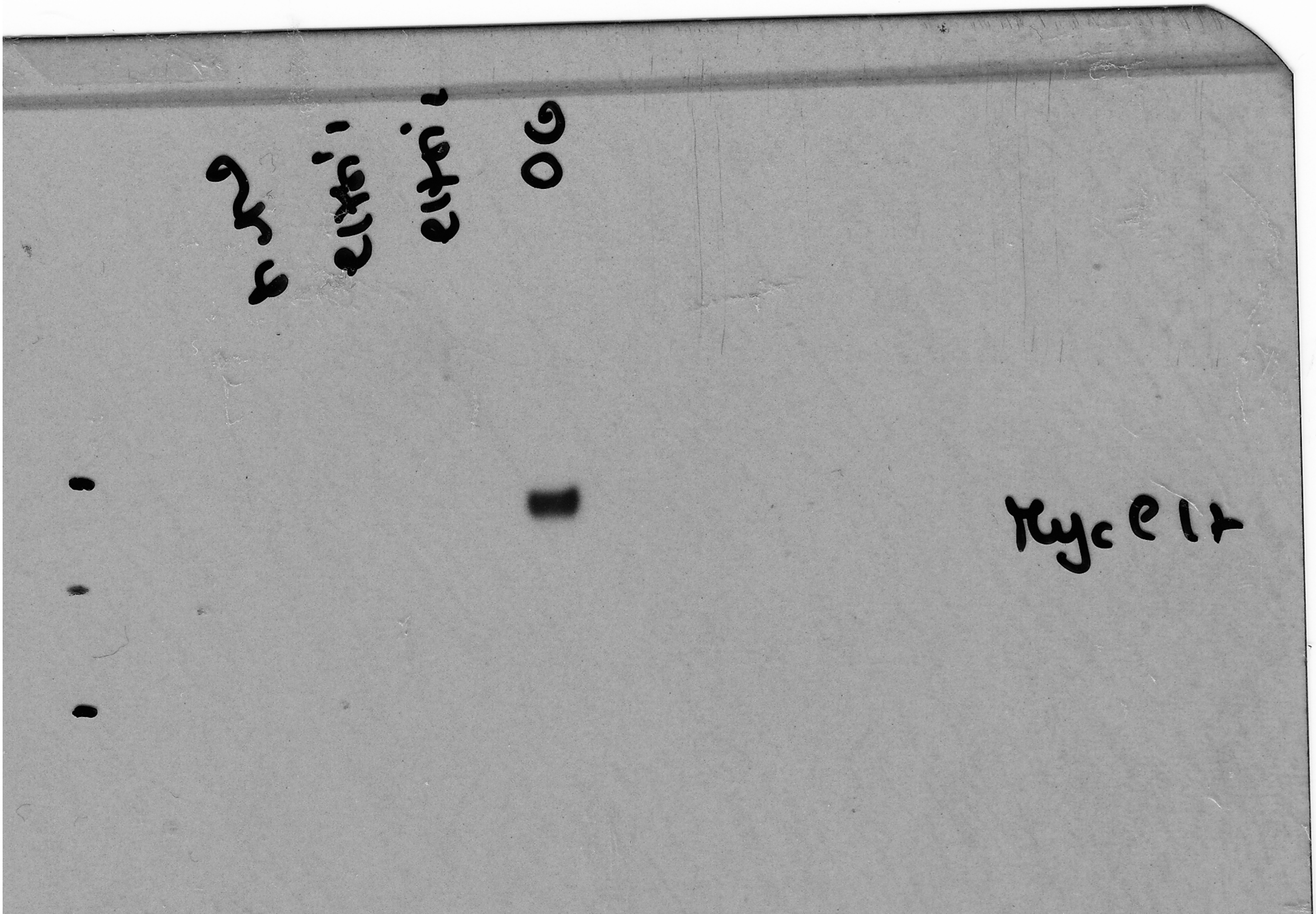

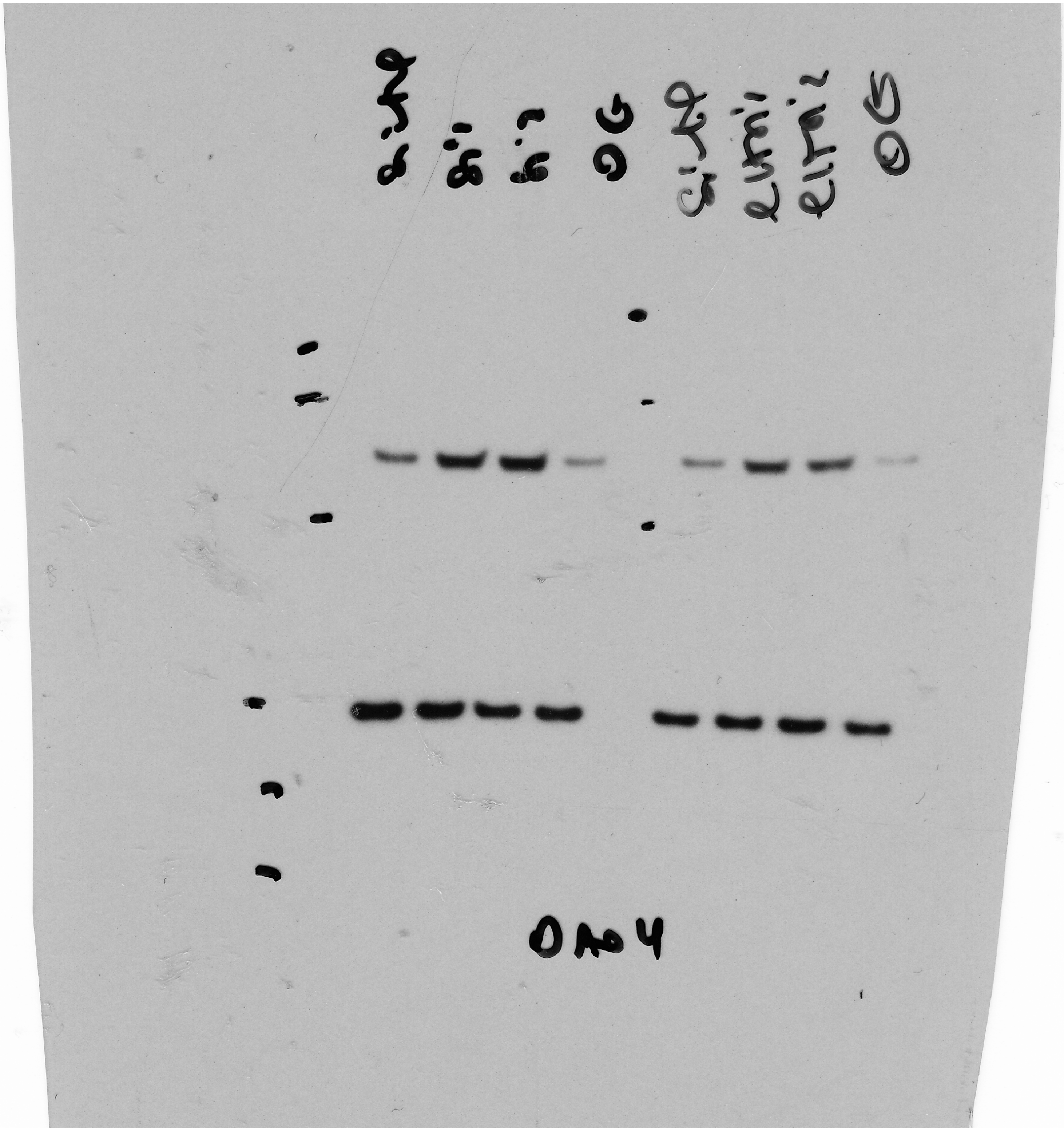
DAY 4
